# Trichothecenes in Cereal Grains – An Update

**DOI:** 10.3390/toxins11110634

**Published:** 2019-10-31

**Authors:** Nora A. Foroud, Danica Baines, Tatiana Y. Gagkaeva, Nehal Thakor, Ana Badea, Barbara Steiner, Maria Bürstmayr, Hermann Bürstmayr

**Affiliations:** 1 Lethbridge Research and Development Centre, Agriculture and Agri-Food Canada, Lethbridge, AB T1J 4B1, Canada; danica.baines@canada.ca; 2 Laboratory of Mycology and Phytopathology, All-Russian Institute of Plant Protection (VIZR), St. Petersburg, Pushkin 196608, Russia; t.gagkaeva@yahoo.com; 3 Department of Chemistry and Biochemistry, University of Lethbridge, Lethbridge, AB T1K 3M4, Canada; nthakor@uleth.ca; 4 Brandon Research and Development Centre, Agriculture and Agri-Food Canada, Brandon, MB R7A 5Y3, Canada; ana.badea@canada.ca; 5 Department of Agrobiotechnology (IFA-Tulln), Institute of Biotechnology in Plant Production, University of Natural Resources and Life Sciences, Vienna (BOKU), Tulln 3430, Austria; barbara.steiner@boku.ac.at (B.S.); maria.buerstmayr@boku.ac.at (M.B.); hermann.buerstmayr@boku.ac.at (H.B.)

**Keywords:** deoxynivalenol, T-2 toxin, *Fusarium*, Fusarium head blight (FHB), wheat, barley, resistance

## Abstract

Trichothecenes are sesquiterpenoid mycotoxins produced by fungi from the order Hypocreales, including members of the *Fusarium* genus that infect cereal grain crops. Different trichothecene-producing *Fusarium* species and strains have different trichothecene chemotypes belonging to the Type A and B class. These fungi cause a disease of small grain cereals, called Fusarium head blight, and their toxins contaminate host tissues. As potent inhibitors of eukaryotic protein synthesis, trichothecenes pose a health risk to human and animal consumers of infected cereal grains. In 2009, Foroud and Eudes published a review of trichothecenes in cereal grains for human consumption. As an update to this review, the work herein provides a comprehensive and multi-disciplinary review of the *Fusarium* trichothecenes covering topics in chemistry and biochemistry, pathogen biology, trichothecene toxicity, molecular mechanisms of resistance or detoxification, genetics of resistance and breeding strategies to reduce their contamination of wheat and barley.

## 1. Introduction: Trichothecene-Producing Fungi and Their Impact on Food and Feed

Trichothecenes are toxic secondary metabolites produced by a variety of fungi from the order Hypocreales, including *Fusarium*, *Myrothecium*, *Stachybotrys*, and *Trichoderma* species [1,2]. This review is focused on the Type A and Type B trichothecenes produced by a group of *Fusarium* species that contaminate food and feed. These species produce and secrete trichothecenes during their interaction with plant hosts [3] while causing a disease of cereals called Fusarium head blight (FHB). *Fusarium* fungi are ascomycetes and are capable of both sexual and asexual reproduction. The teleomorph of many *Fusarium* species belongs to the *Gibberella* genus, although teleomorphs of some *Fusaria* have been reported in other genera, and in many instances, no sexual stage has been identified. That being said, according to the International Code of Botanical Nomenclature for algae, fungi and plants, the naming of pleomorphic fungi (with both asexual and sexual stages) fall under the “One Fungus One Name” rule, and the exclusive use of *Fusarium* is now recommended (since 2013) for all species within this important genus [4].

The *Fusarium* genus has numerous members that are involved in a wide variety of plant diseases [5,6,7,8,9], and while many *Fusarium* species do not produce trichothecenes, those responsible for FHB are primarily trichothecene-producers. The species associated with contamination of food and feed include members of the *F. graminearum* species complex (*Fg* complex), *F. culmorum*, *F. cerealis*, *F. pseudograminearum, F. sporotrichioides*, *F. langsethiae, F. sibiricum*, and *F. poae*. During the time of morphological species identification, members of the *Fg* complex were thought to comprise a single cosmopolitan species. O’Donnell and colleagues employed genealogical concordance phylogenetic species recognition (GCPSR) and in 2000 they reported seven phylogenetic lineages within the *Fg* complex [10]. Today, with current high-throughput multilocus genotyping technology combined with GCPSR and molecular markers, the *Fg* complex is inclusive of 16 phylogenetically distinct cryptic species [11,12,13,14,15]. Regional differences have been reported regarding the occurrence of members within the *Fg* complex, as well as differences in chemotypes and pathogenicity [16,17,18].

*Fusarium* fungi can infect the inflorescence structure of small grain cereals, including wheat, barley, oats and rye under favorable environmental conditions, such as high humidity. Both *Fusarium* ascospores and macroconidia can cause FHB, penetrating the cell wall within 1–2 days post-germination. Over the course of infection, the fungus produces trichothecene toxins that are secreted from the mycelial tip [3]. These toxins are involved in causing necrosis of infected tissues, and have been identified as important factors of aggressiveness [19,20], and in addition to accumulating in the inflorescence and related tissues (e.g., glumes, kernels), they also accumulate in the stems of infected cereals, resulting in contamination of agricultural products [21,22,23]. Toxin accumulation on crop debris is a source of trichothecene contamination in agricultural run-offs, feeding into aquatic environments [24,25].

The main species responsible for FHB are members of the *Fg* complex and *F. culmorum*, but there are regional variations, some of which are discussed in the section on *Fusarium* chemotype distribution. A similar disease occurs in maize, called Fusarium ear blight (FEB) [26], and is also caused by members of the *Fg* complex pathogens as well as a subset of trichothecene non-producing species, such as *F. proliferatum* and *F. verticillioides* (previously *F. moniliforme*) [27]. Cereals and maize are also susceptible to stalk and root rots, and there is an overlap of causative agents for these diseases with those responsible for maladies of the inflorescence. For example, Fusarium crown rot (FCR) of cereals results from infection by *Fg* complex, *F. pseudograminearum*, *F. culmorum,* and *F. cerealis*, and can also result in toxin contamination of the grain [22,23,28,29].

The trichothecenes interact with the 60S ribosomes [30] and are potent inhibitors of protein synthesis in eukaryotes [31,32]. Trichothecene-producing *Fusarium* species are able to protect themselves from the effects of these toxins by secretion through an efflux pump [33]. On the other hand, plants and animals suffer cytotoxic effects when exposed to trichothecenes [34,35,36]. Ingestion of grain contaminated with *Fusarium* trichothecenes has had some devastating impacts within human history. Oral exposure to certain Type A trichothecenes can lead to a fatal condition, known as alimentary toxic aleukia (ATA) [37], where symptoms have been compared with radiation poisoning since the most prominent outcome of this mycotoxicosis is a significant drop in leukocytes [38]. The human impacts of ATA may go as far back as the 5^th^ century B.C.—it has been suggested that the plague of Athens in 430-426 B.C. was an outbreak of ATA [39]. Similarly, symptoms of a reported disease epidemic in New Hampshire in the 1730s are reminiscent of ATA [40].

The most severe recorded outbreaks of ATA occurred within Russian territory between 1932 and 1945, primarily in the regions of Siberia [41,42,43]. Food shortages during this time forced rural people to consume grain and straw that had been left out in the field under snow cover during the winter months. After ingestion of these crops, many people suffered from septic angina and a condition characterized by leukopenia (aleukia), hemorrhagic rash, necrotic ulcers on the lining of the digestive tract and skin, bleeding from the nose, throat, and gums, ultimately leading to death. Similar symptoms were documented in domestic animals following consumption of straw that had been left in the snow until spring.

For many years, no one understood why the rural population suffered from such symptoms, which often led to high human losses (17–50%). The peak of the disease outbreaks was in 1944 when the number of registered cases reached 173 thousand people, of which about 28 thousand died. As a result of this event, significant efforts by a group of scientists who identified and analyzed the mycobiota of a large number of grain samples finally led to the conclusion that ATA symptoms were caused by *F. sporotrichioides*, the most widely occurring microorganism in the samples and causing acute skin toxicity [42]. It was later realized that ATA was directly associated with the presence of Type A trichothecenes, such as T-2 toxin, a mycotoxin that was first isolated in 1968 from contaminated corn associated with bovine mortalities [44].

DON and other Type B trichothecenes are not as potent in mammalian systems compared with T-2 toxin, but can still be lethal at high enough concentrations, as demonstrated in studies with mice [reviewed in 34]. DON is well known for its ability to cause diarrhea and emesis and is sometimes referred to as ‘vomitoxin’. In fact, the ability of DON to elicit vomiting is greater than some of the more potent trichothecenes, such as T-2 toxin [45]. DON and related trichothecenes cause a variety of maladies, including anorexia, feed refusal in livestock, growth retardation, leukocytosis, hemorrhage and adverse effects on reproduction and development [34,46,47,48,49]. Altered neurochemistry and neuron activity have also been reported as an impact of DON exposure [50].

The inadvertent use of DON-contaminated moldy seeds and straw has resulted in numerous cases of food poisoning in people, and in farm animals. FHB epidemics occurred every year in the Russian Far East region from at least 1882. Initially, the symptoms observed in people ingesting food from this tainted grain resembled that of alcohol poisoning: first, a sense of euphoria was observed, later followed by unpredictable behavior, nausea, and vomiting, diarrhea, headaches, abdominal pain, dizziness, and fever. At that time the disease was given the common name “intoxicating bread syndrome”. The rural population had noted a link between health problems and the use of grain and straw from fields where heads were “painted” pink [51]. Printed reports from those times indicate that these health problems rarely resulted in death. The research of mycologists revealed that the fungus *Gibberella saubinetii* (later described *G. zeae*, teleomorph of *F. graminearum*) was the principal causal organism of FHB in the Far East, where environmental conditions are favorable to this disease [52,53]. After chemical identification of DON by Morooka et al. (1972) and Yoshizawa and Morooka (1973) [54,55], it can be inferred that the natural-occurring mycotoxin DON was implicated in these incidences of health problems in both humans and farm animals, which are less severe than the mycotoxicosis from ATA.

Livestock exposure to mycotoxins, including trichothecenes, occurs at all production phases through the consumption of contaminated cereal grains and silages. Exposure can cause direct production losses such as lower milk production in dairy cattle, but also indirect losses due to reduced liver function, immune responses, epithelial barrier function and reproductive capacity [56]. The spotty nature of trichothecene distribution in livestock grains and silages made initial attempts at linking specific trichothecenes with production outcomes largely ineffective. Adding to this issue, a broader range of *Fusarium* species than those responsible for FHB, including the Type A producers, flourish under storage conditions in grain silos and silage pits or bags, whereas the Type B trichothecenes are more commonly isolated from diseased heads resulting in contamination of harvested grain. Cereals and maize infected with *Fusarium* disease also contribute to the proliferation of the fungus during storage in livestock grain-based food. The most effective mechanism to minimize mycotoxin contamination in human and livestock grain-based food is therefore to find ways to prevent or reduce *Fusarium* infection in cereal crops. This can be achieved through improved cultivar resistance brought about by cereal breeding, complemented with changes in agronomic practices that diminish the opportunities for infection. For example, refraining from irrigation during seed-set can reduce or prevent *Fusarium* infection. For post-harvest preservation of livestock grain-based silages, ensilants are added to manage proliferation of *Fusarium* species.

In 2009, Foroud and Eudes published a review of trichothecenes in cereal grains for human consumption [57]. The current manuscript serves as an update of the information therein. New trichothecene chemotypes have emerged, novel insights into the dynamic structure of trichothecenes have been reported, and advances have been made in our understanding of FHB resistance mechanisms—which ultimately leads to reduced trichothecene contamination of cereals.

## 2. Trichothecene Structure, Biosynthesis and Chemotype Distribution

### 2.1. Chemical Structure

**Scheme 1:**
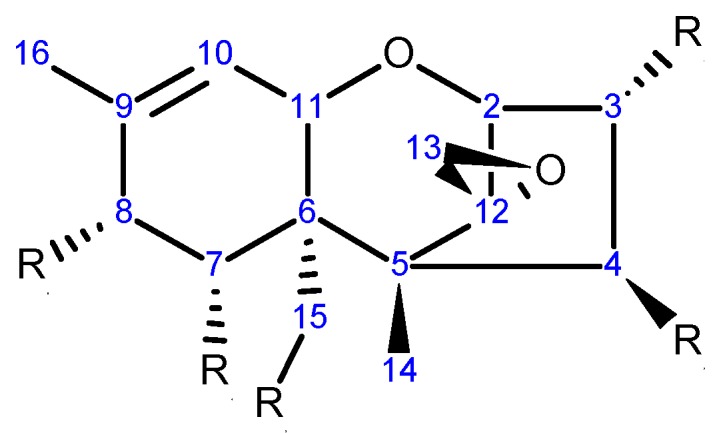
Backbone structure of trichothecene toxins.

Trichothecenes are sesquiterpenoid compounds composed of multiple fused rings [58]. The backbone has a central cyclohexene core (A-ring) fused to a tetrahydropyran (B-ring), which in turn is fused to a cyclopentyl moiety (C-ring) at C_2_ and C_5_. In addition, an epoxide attached to C_12_ is known to be essential for toxicity [59] (Scheme 1). More than 200 trichothecenes have been identified, differing in their substitution at five positions along the backbone (C_3_, C_4_, C_7_, C_8_, and C_15_) [60]. Substituents of the *Fusarium* trichothecenes are typical: hydrogen (-H), hydroxyl (-OH), ester-linked acetyl (-OC(=O)CH_3_) or ester-linked isovalerate (-OC(=O)CH_2_CH(CH_3_)_2_) groups. There are four trichothecene classes (Types A-D; for a review see McCormick et al. [61]) and the *Fusarium* species produce members of the Type A and/or B classes. A list of important *Fusarium* trichothecenes and their functional groups is presented in Table 1.

Type B trichothecenes can be distinguished from Type A by a ketone (=O) at C_8_ [61]. T-2 toxin and diacetoxyscirpenol (DAS) are examples of Type A trichothecenes; T-2 toxin carries an *O*-isovalerate group at C_8_, whereas DAS does is unsubstituted at this position. Both T-2 toxin and DAS have C_3_ hydroxyls and *O*-acetyl groups at C_4_ and C_15_. 4′-hydroxy-T-2 toxin (HT-2 toxin) differs from T-2 toxin by substitution of the *O*-acetyl vs. *O*-hydroxyl at C_4_. In recent years, an emergent group of Type A trichothecenes has been reported in North America: NX-2 and NX-3 [16,62,63]. These mycotoxins are similar in structure to the Type B trichothecenes 4-deoxy-nivalenol (DON) and its acetylated derivative 3-*O*-acteyl-DON (3-ADON), respectively, differing only in the presence of the C_8_ ketone. Nivalenol (NIV) is a Type B trichothecene, hydroxylated at all positions, excepting of course the C_8_ ketone. Acetylated derivatives of NIV also exist, including 4-*O*-acetyl-NIV (4-ANIV).

### 2.2. Trichothecene Biosynthesis

**Scheme 2:**
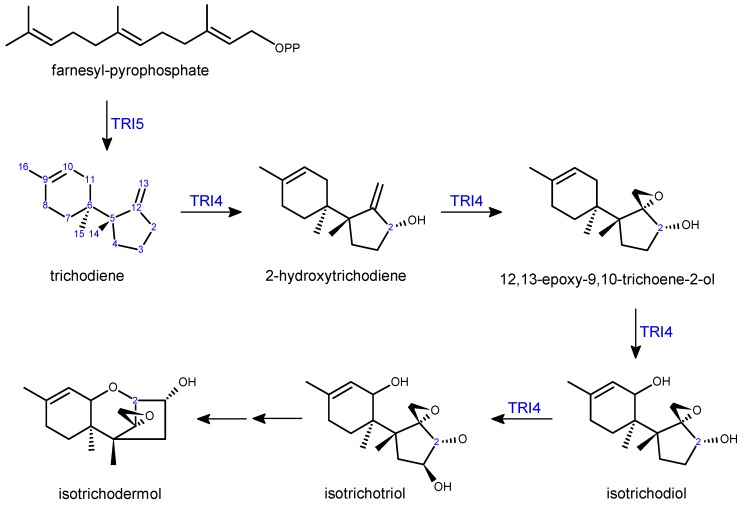
First steps of trichothecene biosynthesis. Trichodiene is synthesized from the secondary metabolite farnesyl pyrophosphate through the activity of trichodiene synthases, encoded by the *TRI5* gene. TRI4 catalyzes a series of hydroxylations and an epoxidation step to yield isotrichotriol. Isotrichotriol undergoes non-enzymatic isomerization steps to yield isotrichodermol.

Trichothecene biosynthesis and regulatory genes (*TRI* genes), can be found in clusters on the genome; this is the case for *Fusarium* species as well as other trichothecene producing genera [64,65,66,67]. The core cluster in *Fusarium* includes the majority of the *TRI* genes [68], while four remaining genes are located at three different loci: the TRI1-TRI16 two gene cluster [69] and two independent loci for *TRI101* [70] and *TRI15* [71]. A list of *Fusarium TRI* genes with a brief description of the function is presented in Table 2. Trichothecene biosynthesis is initiated by a sesquiterpene cyclase which yields trichodiene from its substrate, farnesyl pyrophosphate [72,73] (Scheme 2). The enzyme, trichodiene synthase (previously described as Tox5), is encoded by the *TRI5* gene and was cloned from *F. sporotrichioides* in 1989 [74], and *TRI5* genetic disruption mutants generated in a number of *Fusarium* species have been utilized to investigate the role of trichothecenes in plant pathogenesis [19,20,75]. A multifunctional cytochrome P450 monooxygenase, encoded by *TRI4*, catalyzes the next four steps in the pathway (Scheme 2): C_2_ hydroxylation [76], C_12,13_ epoxidation, followed by hydroxylation at C_11_ and C_3_ [77,78]. The product, isotrichotriol, undergoes two non-enzymatic isomerization steps [79] forming the central ring of isotrichodermol, which consists of the base trichothecene skeleton structure with a C_3_ hydroxyl group.

**Scheme 3:**
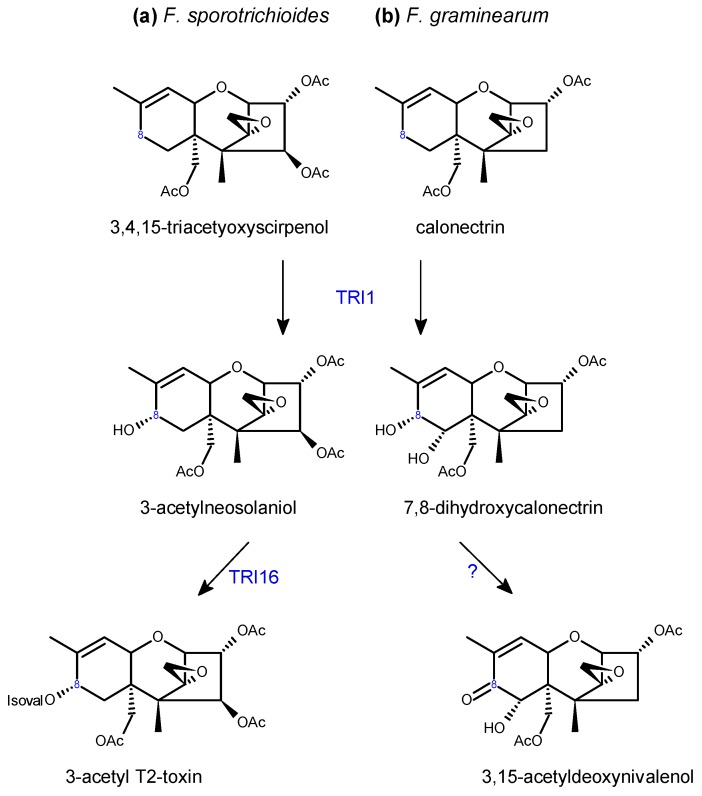
TRI1 catalyzes C_8_ hydroxylation in (**a**) *F. sporotrichioides* and (**b**) *F. graminearum*. TRI16 activity then catalyzes the esterification of isovalerate (isoval) at the C_8_ hydroxyl in *F. sporotrichioides*. In *F. graminearum*, TRI1-mediated hydroxylation occurs at both C_7_ and C_8_, followed by an oxidation reaction to form the C8 ketone group. No specific gene has been linked with the formation of the C_8_ ketone.

Sequence analyses of *TRI* genes are used to predict trichothecene chemotypes of different *Fusarium* strains, although biochemical analysis is necessary to confirm the production of specific mycotoxins [102]. The *TRI1-TRI16* cluster is responsible for coordinating the functional group at C_8_ and is therefore involved in differentiating Type A from Type B trichothecenes (Scheme 3). *TRI1* is required for oxygenation at C_8_, by catalyzing the addition of a hydroxyl group [82], in *F. graminearum* this group can be esterified by TRI16 to an isovalerate group, as found in T-2 toxin and HT-2 toxin, whereas the *TRI16* gene is non-functional in *F. graminearum* [69]. In *F. graminearum*, allelic variations in *TRI1* differentiate NX vs. DON chemotypes [62].

**Scheme 4:**
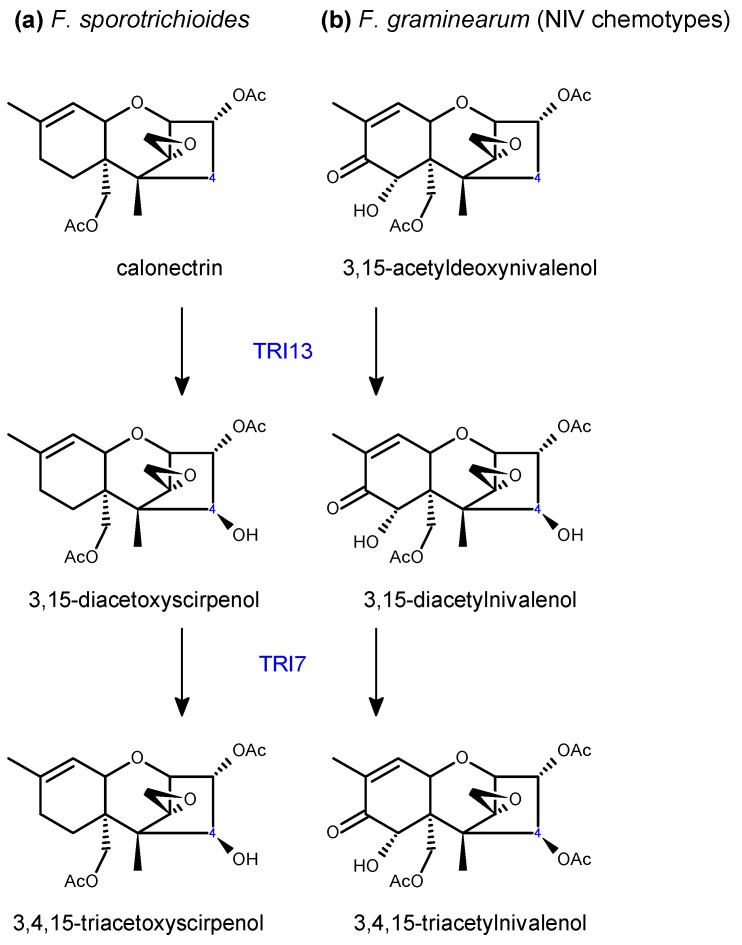
TRI13 and TRI7 catalyze hydroxylation and acetylation of C_4_, respectively in (**a**) *F. sporotrichioides* and (**b**) NIV-producing *F. graminearum*.

**Scheme 5:**
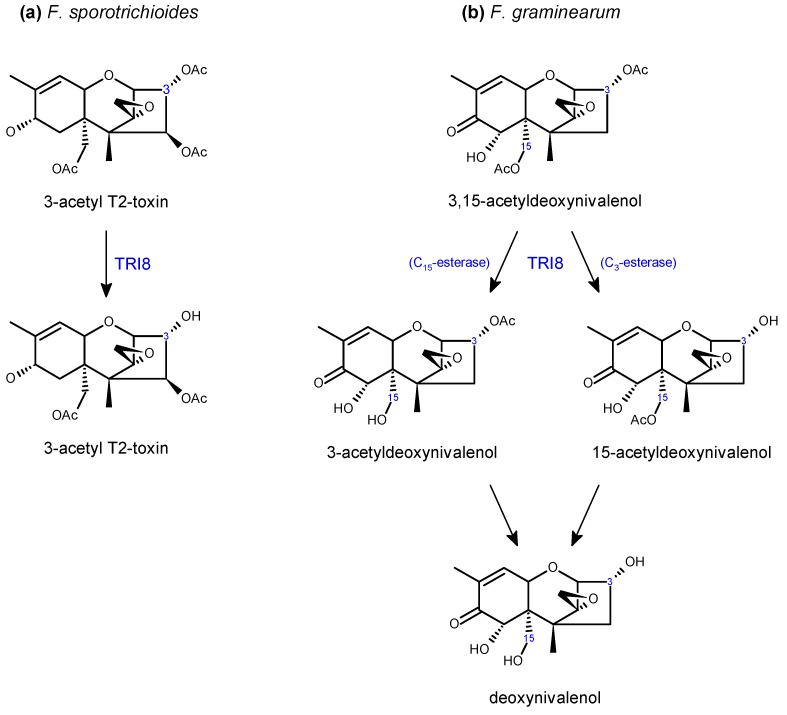
*TRI8* encodes a C_3_ or C_15_ esterase. (**a**) C_15_ esterase activity is required for T-2 toxin production in *F. sporotrichioides*, whereas (**b**) C_15_ or C_3_ specificities are encoded in *F. graminearum* which differentiates 3-acetyldeoxynivalenol and 15-acetyldeoxynivalenol chemotypes, respectively.

Tri13 and Tri7 are required for oxygenation and acetylation, respectively, at C_4_, playing key roles in NIV production in *F. graminearum* [86,91] and T-2 toxin production in *F. sporotrichioides* [91] (Scheme 4). Both genes have lost functionality in the DON chemotypes, hence the absence of the C_4_ hydroxyl in this toxin. DON chemotypes also produce acetylated derivatives, 3-ADON or 15-ADON. This is executed by C_3_ or C_15_ de-acetylation of 3,15-ADON, which is originally acetylated by *TRI101* and *TRI3* (Scheme 5). Allelic variations of *FgTRI8* determine stereo-specificity of the encoded esterase, thereby determining whether the DON chemotype in a given *F. graminearum* strain produces 3-ADON or 15-ADON [89] (Scheme 5). In *F. sporotrichioides*, *FsTRI8* is a C_3_ esterase necessary for the biosynthesis of T-2 toxin, which carries a hydroxyl group at this position [88] (Scheme 5).

Three transcription factors have been identified in the core gene cluster, *TRI6*, *TRI10,* and *TRI15*. TRI6 is a Cys_2_His_2_ zinc finger protein [94] that binds most promoter regions within the trichothecene gene clusters and positively regulates its own expression [95]. ChIP-Seq revealed that TRI6 interacts with numerous genes involved in cellular metabolism and signal transduction, in addition to the *TRI* genes [95]. The binding motif was earlier predicted as YNAGGCC [103]. However, electromobility shift assays using the TRI6 protein revealed that this transcription factor binds to a GTGA/TCAC motif (GTGA-X_6-8_-TCAC), and it was suggested that the YNAGGCC motif might instead be recognized by a TRI6-protein complex [95]. *TRI6* deletion mutants are unable to produce trichothecenes and have reduced pathogenicity in FHB, FCR and Fusarium root rot (FRR) [104,105]. While not required for *TRI10* expression, TRI6 may nonetheless be involved in its regulation. Tag et al. [97] observed that TRI10 upregulates *TRI6* expression and suggested that TRI6 may then downregulate *TRI10*. TRI10 regulates the expression of numerous *TRI* genes [96,97], and the effect of *TRI10* deletion results in reduced trichothecene expression but does not eliminate it entirely [97]. Both *TRI6* and *TRI10* deletion mutants show significant reductions in pathogenesis [106]. TRI15 is a Cys_2_His_2_ zinc finger protein that does not appear to be necessary for T-2 toxin production in *F. sporotrichioides* and is instead thought to be a negative regulator of trichothecene biosynthesis [71]. The gene was cloned and characterized by *F. sporotrichioides*, and a homologous sequence identified in *F. graminearum* [71].

The biological activities of TRI9 and TRI14 are unclear, meanwhile, the encoding genes are both upregulated alongside other *TRI* genes during trichothecene biosynthesis in *F. graminearum* [107], and also during infection of wheat spikes [108], suggesting a biological function related to trichothecene biosynthesis and/or pathogenesis. In fact, while its activity remains unclear, *TRI14* was found to influence disease as it is required for DON production during *F. graminearum* interaction with wheat [100].

### 2.3. Fusarium Species and Chemotype Distribution

Trichothecene-producing *Fusarium* species synthesize numerous mycotoxins, including multiple trichothecenes along with different classes of toxins such as zearalenone, butenolides, and gramillins [109,110,111,112,113]. It is also worth mentioning that there are some *Fusarium* species responsible for FHB that do not produce trichothecenes, for example, *F. avenaceum*, an important FHB pathogen in Europe and North America, produces instead beauvericin, enniatins and moniliformin [114,115,116]. The maize pathogen involved in FEB, *F. verticillioides* produces fumonisins and moniliformin [27,117].

Toxin compositions are both species and strain-dependent and also vary according to the nutritional status of the grains or substrate, nevertheless one or two trichothecenes typically dominate the chemotype. The global distribution of *Fusarium* species and chemotypes within a species is heavily influenced by climatic factors. In addition, there are species- or chemotype-specific host preferences among different strains. That said, the main species involved in FHB throughout the world are members of the *Fg* complex and *F. culmorum* [26,118,119,120,121,122], where the latter tends to be more prolific in cooler regions than the former [123]. With exception of the recently identified NX toxins from *F. graminearum* strains in North America [62], the *Fg* species complex are Type B trichothecene producers. The trichothecene DON is often the most abundant and frequently detected mycotoxin in cereal grains [26,115,120,121]. DON is also frequently isolated from maize ears, although zearalenone (ZEA) is an important class of toxins associated with this crop and tends to be particularly problematic during storage [26].

DON chemotypes produce acetylated derivatives of DON (e.g., 3-ADON or 15-ADON) that also accumulate in the grain. Historically, both 3-ADON and 15-ADON chemotypes were isolated throughout Eurasia, while 15-ADON chemotypes were more abundant in North America [124,125,126]; however, both chemotypes have been identified on both continents. A survey of 29 European countries from 2000 to 2013 revealed that the 15-ADON is the dominant chemotype among *F. graminearum* strains, representing 82.9% of the population, whereas 3-ADON was 13.6% were 3-ADON chemotypes [127]. A combination of genotyping and chemotyping of *F. graminearum* shows that the 15-ADON types dominate in southern and central Europe [127,128,129,130], whereas 3-ADON is more prevalent in the northwestern regions [14,131,132,133]. Recently the 15-ADON type was introduced in Norway [132] and Denmark [133].

In North America, the 3-ADON producing strains were not endemic, having been introduced sometime in the 1990s, but the proportion of 3-ADON chemotypes identified in Canada and the United States has been increasing over the years [134]. The reason for this shift is uncertain as there is no clear evidence that the 3-ADON types have a fitness advantage over the 15-ADON strains [135]. In controlled environment experiments, Gilbert et al. [136] observed that the 3-ADON chemotypes isolated in Canada grew better at higher temperatures (28 °C) compared with 15-ADON chemotypes (18–22 °C). However, data collected from field surveys did not support a correlation in temperature and the ratio of 3-ADON to 15-ADON producing strains.

The 3-ADON chemotypes commonly produce more DON in vitro than the 15-ADON chemotypes [134], and this difference has been reflected in the abundance of toxin accumulated in wheat and barley heads inoculated with either chemotype [137,138,139,140,141]. Some emergent 15-ADON strains have also been identified in North America, with genetic similarities to the 3-ADON chemotypes. These emergent strains also produce higher levels of toxin [134]. Differences in pathogenicity have also been reported in some greenhouse experiments between the 3- and 15-ADON chemotypes, where the 3-ADON chemotypes can be more aggressive in some genotypes [139,141] or inoculation methods [142] in wheat. Similar observations were made for the emergent 15-ADON strains [139]. It is not known whether the observed differences in aggressiveness are related to the higher abundance of toxin produced by the 3-ADON and emergent 15-ADON chemotypes. Walkowiak et al. [143] studied the interaction of a 3-ADON *Fg* strain with a 15-ADON strain and compared the genomes of both strains. They identified 97% base pair alignment, and the 3% variation corresponded to SNPs and indels predicted to be involved in virulence and also observed 13% differential gene expression in vitro. The same group later published the pan-genome of ten strains from the *Fg* complex [144]. Among these, four 15-ADON and two 3-ADON producing strains of *F. graminearum sensu stricto* were compared and a 3.6% variation was observed among these genomes, where the two chemotypes formed genetically distinct clades. The genetic differences between these groups are thought to contribute to differences in aggressiveness.

NIV chemotypes do not produce DON, and their *in planta* toxin production capacity tends to be lower than that of DON chemotypes [139,145,146]. NIV is less phytotoxic than DON and this chemotype is typically less aggressive in wheat and rye [139,147]. DON chemotypes generally dominate the *Fusarium* populations isolated from infected cereals. In a 7-year survey of European countries published in 2016 by Pasquali et al. [127], only 3.5% of the *F. graminearum* isolates were NIV chemotypes and the remaining 96.5% where DON producers. Most of the NIV chemotypes were found in Western Europe [127], and while DON producers are still dominant chemotype in this and other regions, there are geographic niches where other chemotypes take over. For example, NIV chemotypes represent nearly 80% of the populations identified in the state of Louisiana, United States [146], and NIV genotypes are predominant in different regions of northern Iran [148,149].

Though less abundant, the identification of NX-2/NX-3 chemotypes in North America has attracted a fair amount of interest. *F. graminearum* was previously thought to only produce Type B trichothecenes, and in North America, the Type B producers responsible for FHB are divided into two main groups: NA1 are represented primarily by the endemic 15-ADON producing strains, and NA2 the introduced 3-ADON producing *F. graminearum* strains [150]. When first isolated, the NX producers were thought to be 3-ADON producing strains based on genetic analyses, but unlike the 3-ADON chemotypes this group did not appear to produce trichothecenes in inoculated wheat spikes. These strains were identified from surveys of wheat, as well as non-agricultural grasses, in the northern United States between 2003 and 2006 by Gale et al. [151]. It was later determined that these ‘trichothecene non-producers’ in fact produce the Type A trichothecenes, NX-2 and NX-3 [62], and they have a broad host range having been isolated from wheat, barley, oats and maize [16,63,152]. Analyses of isolates collected around the globe indicate that these strains are endemic to North America and have only been identified in the northern United States and southern regions of Canada [16,152]. To date, they tend to occur at low frequencies, accounting for approximately 2% of the *F. graminearum* population within their known geographic regions [152]. That being said, there is some variation in their distribution in different areas and also on different crops within a region. For example, in the analysis carried out by Kelly et al. [152] the NX-2 strains represented 6.0% of the isolates collected from Québec barley and 3.3% of those isolated from oats in Manitoba. Most recently, it was determined that the frequency of the NX-2 genotype is nearly 20% in northeastern New York. In this case the authors, Lofgren et al. [153], analyzed the *TRI1* allele of 133 samples that had previously been genotyped as 3-ADON and found that close to 50% of these were, in fact, NX-2 genotypes. The NX-2 chemotype for eight of these isolates was verified by GC-MS. This is one example that illustrates the importance of biochemical analysis in the assignment of chemotypes. Genotyping offers higher throughput and is more accessible to many labs, but the assignment of “chemotype” should only be made if chemical analysis has been done, while “genotype” can be used where trichothecene chemotypes are predicted based on genetic analyses alone [102].

The origins of the NX genotypes are unknown, except that they are by all accounts endemic to North America [152], they share genetic similarities with both NA1 (based on tandem repeat markers) [16] and NA2 (based on restriction site polymorphism) [154]. Kelly and Ward [150] determined that the NX genotypes belong to a distinct population, which they described as the NA3 population.

Though generally less pathogenic compared with *F. graminearum* and *F. culmorum* [155], members of Type A trichothecene producing *F. sporotrichioides* and *F. langsethiae* are also causative agents in FHB [156], and their toxins, T-2 toxin, and HT-2 toxin are frequently isolated from infected heads in some temperate regions of Europe [157,158,159,160]. Indeed, many of the Type A producing species proliferate in cooler climates. A two-year survey in northern Spain showed a preference for Type A producers in the cooler regions and Type B in the warmer areas [159]. While this class of trichothecene typically occurs at lower frequencies and concentrations than DON, data collected from Nordic countries, Central Europe and the United Kingdom, indicate that their occurrence is on the rise [161,162,163,164,165]. It should be noted that T-2 and HT-2 toxins are more frequently isolated from oats and barley than in wheat or other cereals [163,166,167]. In a study of United Kingdom oats sampled between the years of 2002 and 2005, Edwards [163] observed a higher mean concentration of T-2 and HT-2 toxin than had previously been reported in any cereal class worldwide.

Many attribute the increase in T-2 and HT-2 toxins to higher infection frequencies by *F. langsethiae* [168,169,170]. This species, originally described as “powdery *F. poae*” [171], tends to grow symptomless on oats but accumulates high levels of toxins [172], where it has been suggested to have some degree of host preference over wheat [173]. *F. langsethiae* is considered the most important T-2 and HT-2 toxin producer [161,174,175] and is currently detected in nearly all territories in the northern and southern regions of Europe [176,177,178,179,180]. Apart from a single isolate found in western Siberia, *F. langsethiae* has not been detected outside of Europe [181].

Although not considered a major problem in North America, Type A producer *F. sporotrichioides* has been identified in Canada and the United States [182,183]. For example, Tittlemier et al. [116] identified these species in Canadian durum wheat and isolated both T-2 toxin and HT-2 toxin from the grain. This indicates that these species are also present in North America, and in the right environment or storage conditions, they have the potential to increase their impact in the Americas. The tendency of the Type A producers to proliferate in cooler climates may explain why many of these species are often found on grain in storage or over-wintered in the field.

## 3. Trichothecene Toxicity

### 3.1. Disruption of Eukaryotic Protein Synthesis

The main target of trichothecene toxicity is the eukaryotic ribosome, as first determined by Ueno et al. in the late 1960s [184], where it binds and disrupts protein synthesis through its interaction with the peptidyl transferase center [30]. Other cellular impacts have been observed on nucleic acid biosynthesis, mitosis and membrane/organelle integrity (reviewed in [185])—but these are likely all secondary effects of protein synthesis inhibition and ribotoxic stress, and direct interaction between the trichothecenes and cellular components has only been reported for the ribosome. Ueno et al. [184] first reported NIV-mediated inhibition of protein synthesis in rabbit reticulocyte whole cells and cell-free lysates in 1968. Numerous studies on the effect of various trichothecenes on ribosome activity in mammalian cell extracts followed in the 1970s, with considerable attention on identifying the mode of inhibition [59,186,187,188,189,190,191,192,193]. While it was clear that the trichothecenes interfered with activity at the peptidyl transferase center, it was not known whether inhibition occurred at initiation (I), peptide elongation (E) or termination (T). Polyribosome shift assays suggested that specific trichothecenes inhibited different stages of translation (Table 3), although consensus was not achieved in many instances. For example, it was reported that trichodermin inhibits translation termination in vivo in both yeast [188] and HeLa cells [190], and also in vitro in rabbit reticulocyte lysates [190]. Another study in H-HeLa suggested that trichodermin exhibited both E-/T-type inhibitions [186]. Meanwhile, Carter et al. [192] were unable to replicate T-type inhibition in rabbit reticulocyte lysates and proposed that trichodermin is instead an E-type inhibitor. In 2014, the crystal structure of the yeast ribosome was resolved up to 2.9 Å in the presence of different protein synthesis inhibitors, and all three of the trichothecenes screened, DON, T-2 toxin and Verracurin A (a type D trichothecene), were bound to the A-site of the peptidyl transferase center [30]. These results indicate that the trichothecenes would affect peptide bond formation during elongation, suggesting that the trichothecenes are E-type translation inhibitors.

### 3.2. Structure-Activity Relationship: Importance of the 12,13-Epoxide

**Scheme 6:**
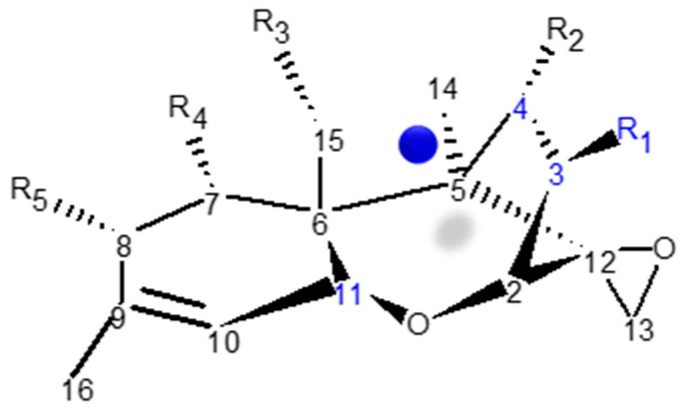
A schematic representation of the trichothecene core structure with a bound water molecule (depicted as a blue sphere) situated over the tetrahydropyran ring. In both T-2 toxin and DON, proton exchange occurs between the 3′OH at position R_1_, hydrogen-bonding also occurs with protons at C_3_, C_4_ and C_11_ [196,197].

The 12,13-epoxide group is known to be essential for trichothecene toxicity [194,195]. Epoxides are highly reactive groups, although they are unusually stable in the trichothecene structure. It was surprising that in the trichothecene-bound ribosome crystal structure, no direct interaction was reported between the epoxide ring and ribosomal components [30]. Foroud et al. [196] proposed that the epoxide ring is required for structural stability to the trichothecene skeleton, thereby rendering it essential for toxicity. When investigating the structure of trichothecenes in various hydroscopic solvents, a water molecule is always present [196,197,198]. The water is bound to a pocket formed between the B- and C-rings and this pocket are fairly rigid with limited torsional flexibility as a result of tension imparted by the epoxide ring, which pulls C_12_ down to form the base of the pocket (Scheme 6). In DON, the water forms a bridge between C_3_ and C_15_ through interactions with the hydroxyl groups at these positions [196]. Similar interactions have been observed for T-2 toxin, where the C_3_ hydroxyl undergoes proton exchange with one of the hydrogens on the water. By contrast with DON, T-2 toxin has an acetyl function at C_15_, preventing the formation of the water bridge. The conformation observed for water-bound T-2 toxin and DON is similar to what is found in the crystal structure presented by Garreau de Loubresse et al. [30]. In their interaction with the ribosome, the binding pocket is positioned in the vicinity of a magnesium ion [30], which appears to occupy similar spaces as the water molecule in the solvent structure of DON and T-2 toxin [196,197]. Mg^2+^ affects the assembly and stability of the ribosome. The local conformation in the peptidyl transferase center would be disrupted in the absence of Mg^2+^, and hence the peptidyl transferase activity would be inhibited. DON may sequester Mg^2+^ in the peptidyl transferase center and thereby inhibit peptidyl transferase activity, which results in inhibition of protein synthesis [199]. Based on these data, Foroud et al. [196] hypothesized the epoxide ring serves to stabilize the structure of the binding pocket to enable interactions with water or other molecules, such as magnesium, thereby disrupting peptidyl transferase activity during translation elongation.

### 3.3. Structure-Activity Relationship: Influence of Substitution Patterns to the Trichothecene Core

**Figure 1:**
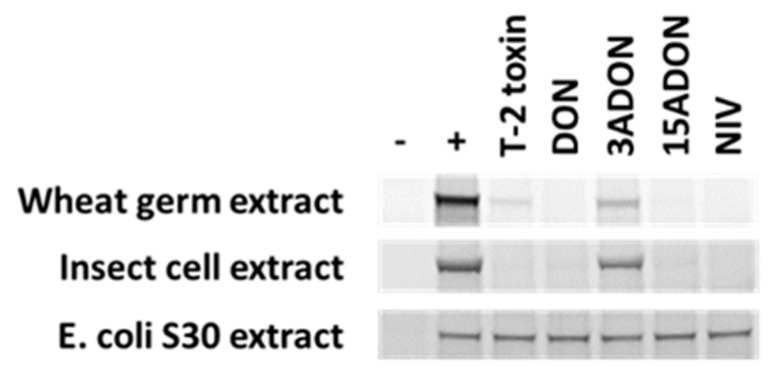
Translation of the luciferase mRNA in three cell-free translation systems (Promega), with FluoroTect™ Green_Lys_ in vitro Translation Labeling System, according to manufacturer’s instructions, and in the presence or absence of different trichothecenes at 20 µM concentration.

The substitution pattern at the C_3_, C_4,_ and C_15_ is thought to affect the nature of the interaction with water [196]. For example, modifications at C_3_ may affect the affinity of water binding in DON. Certain modifications of C_3_, such as acetylation [62,200,201,202] or glucosylation [203], have been found to minimize or disrupt cytotoxicity. These modifications have also been shown to limit or prevent the inhibition of protein translation [62,200,203,204]. Interestingly, there are instances where C_3_ acetylation does not appear to reduce phytotoxicity. For example, Desjardins et al. [205] evaluated the phytotoxicity of 24 trichothecenes on *Arabidopsis* leaves, eight of which were acetylated at C_3_. When comparing each of these eight compounds with their C_3_ hydroxylated counterparts, only three showed reduced phytotoxicity and, in one case, toxicity was increased. These observations may be a direct result of the toxin’s ability to inhibit protein synthesis but could also be related to cellular uptake or processing of DON or 3-ADON *in planta* [35]. It has in fact been demonstrated that wheat can convert acetylated DONs to DON, for example, 3-ADON can be de-acetylated *in planta* [206]. This might explain limited inhibitory effects of 3-ADON on in vitro translation of luciferase in eukaryotic cell extracts, including wheat germ (Figure 1), despite the apparent lack of difference in phytotoxicity between 3-ADON and DON in wheat [207] or *Arabidopsis* [205].

The effect of other substitution patterns has also been investigated to identify structure-activity relationships, though it is important to note that there are host-dependent variations in toxicity. NIV, which differs from DON only in the absence of a hydroxyl group at C_4_, seems to be less phytotoxic than DON [205,207], but tends to be more toxic in mammalian systems compared with the latter [34,208]. The most important Type A trichothecenes, T-2 toxin, HT-2 toxin, and DAS, are much more potent in mammalian systems than the Type B members, including DON and NIV [209]. An interesting exception to this trend was observed for NX-3 and NX-2, which have shown similar toxicity as their Type B counterparts, DON and 3-ADON, respectively, in both in vitro translation reactions with rabbit or wheat ribosomes and in phytotoxicity assays with *Chlamydomonas reinhardtii* [201]. Thompson and Wannemacher [204] compared 19 trichothecenes and observed a trend of increased potency for many Type A trichothecenes in their ability to inhibit protein synthesis in mammalian cell lines compared with the Type B group. Similar trends have been observed in toxicity on some plants, such as *Arabidopsis* and *C*. *reinhardtii* [201,205]. By contrast, when comparing phytotoxicity, T-2 toxin, HT-2 toxin, and DAS had limited effects on wheat coleoptile inhibition, especially when compared with DON [207]. This may be directly related to differences in protein synthesis inhibition in wheat: T-2 toxin was only able to partially inhibit protein translation in wheat germ extracts at a high concentration (20 µM), which for DON led to complete inhibition (Figure 1). That being said, protein translation inhibition does not always reflect toxicity within the organism, as has been described above for 3-ADON in wheat, and also reported in mammalian systems [204]. Thus, while the different substitution patterns seem to affect trichothecene toxicity, and some important trends have been observed, there are organism-specific differences both in cytotoxicity and in their ability to inhibit protein synthesis, and protein synthesis inhibition does not always reflect the level of toxicity in the organism. The ribosome structure is highly conserved across eukaryotes, but differences in protein synthesis inhibition are nonetheless observed among trichothecenes when comparing organisms from different Kingdoms (e.g., Figure 1). Due to the conserved nature of the ribosomes, it would stand to reason that there may be limited divergence within phylogenetic Kingdoms. Thus, the differences observed in cytotoxicity within a Kingdom are perhaps more likely related differences in cellular uptake and/or metabolic processing. In fact, when comparing different treatment methods within an animal model, differences have been observed in uptake, processing, and toxicity [34].

## 4. Molecular Mechanisms of Trichothecene Resistance

### 4.1. Trichothecene Resistance at the Ribosome

Owing to the ubiquitous and essential activities of the ribosome, its structure is highly conserved within phylogenetic domains, making it a reliable target for pathogens. The ribosome can resist the effect of trichothecenes if a point mutation occurs at the tryptophan (W) residue within the highly conserved W-finger of Ribosomal Protein L3 (RPL3) [210,211]. Mutations at this site also confer resistance to other “antibiotics” [e.g., anisomycin, 211], that bind the A-site of the peptidyl transferase center. The W-finger projects directly into this site, and the RPL3 protein coordinates aa-tRNA accommodation, peptidyl transfer and translocation steps during protein translation [212]. Only a few substitutions of the tryptophan in this position are viable, including substitutions with other aromatic residues, or with cysteine or arginine. Studies in yeast show that the W255C substitution increases affinity to aa-tRNA, and while this mutation is non-lethal, it does result in reduced translation efficiency [213].

It is not known whether W-finger mutations occur as a natural resistance mechanism in plants, although in vitro translation assays demonstrated that ribosomes isolated from the FHB-resistant cultivar, Frontana, exhibit a higher tolerance to DON than those isolated from the susceptible cultivar Cassavant [214]. Transgenic expression of modified *RPL3* has had mixed results. It was determined that the expression of the tomato RPL3 gene with a modified W-finger (W258C) had a minimal effect on the ability of transgenic tobacco to tolerate DON [210]. Additionally, transgenic plants were found to preferentially utilize the wild-type RPL3 (except at sub-lethal concentrations of DON) and these observations were corroborated using yeast transformants. On the other hand, transgenic expression of a W258C modified rice *RPL3* conferred DON tolerance in tobacco protoplasts [215], as well as FEB resistance in maize [216]. It has also been suggested by Di et al. [217] that expression of a truncated *RPL3* from yeast could reduce DON toxicity in cereals by drawing the toxin away from the functional wild-type RPL3. The authors expressed truncated yeast RPL3 (ΔL3) in wheat and found that, in addition to an increase in the expression of the endogenous wheat RPL3 gene, transgenic plants displayed a reduction in FHB symptoms as well as DON accumulation.

In a similar study, the same group had earlier reported that co-expression of ΔL3 together with a Pokeweed Antiviral Protein (PAP) in tobacco cells resulted in increased expression of the host *RPL3* gene, and this was coincident with improved DON tolerance [218]. In this context, it is interesting that the up-regulation of different components of the ribosome has been observed directly in response to exogenous DON applications in wheat. In a transcriptomics study by Foroud et al. [219], DON induced higher expression of ribosomal components, but only in the resistant genotypes, which incidentally were generated through a process that provides selection pressure for trichothecene resistance. There are many potential explanations for this up-regulation, though at this stage only speculation can be offered, and it is also unknown whether there is a direct link with this expression pattern and resistance in these genotypes. It should be noted, however, that changes in ribosomal gene expression have been reported in different plant-pathogen interactions (e.g., [220]), and importantly, according to the ‘ribosome filter hypothesis’ alterations in ribosomal composition or specialized ribosomes are thought to bias incorporation of specific mRNAs to translation [221,222]. Thus, the filter hypothesis offers the possibility that the reported DON-induced changes in ribosomal gene expression might coordinate defense-related translation rather than conferring ribosomal resistance to the toxin.

### 4.2. Trichothecene Efflux

Since trichothecenes inhibit the activity of eukaryotic ribosomes, as eukarya the trichothecene producing fungi require a mechanism to deal with toxins that interfere with their own protein synthesis machinery. The ribosomes of *Fusarium* species are not known to directly resist the effects of trichothecenes, but these fungi do rely on toxin efflux through the *TRI12* gene product (Table 3). Efflux is a common mechanism of self-protection employed by toxin-producing microorganisms, often through the channels of integral membrane proteins of the ATP-binding cassette (ABC) superfamily or major facilitator superfamily (MFS) transporter classes [223,224], and TRI12 is an MFS transporter localized at the plasma membrane [98]. The gene was first cloned in 1999 from *F. sporotrichioides* by Alexander and colleagues [33], and genetic disruption thereof reduced radial growth on complex medium compared with the wild-type progenitor strain. This effect was further exasperated by the addition of exogenous DAS. The authors also observed a substantial reduction in trichothecene accumulation in liquid culture; only a small amount of T-2 toxin was detected [33]. When disrupting the orthologous gene in a DON producing *F. graminearum* strain, PH-1, Menke et al. [98] observed reduced accumulation of trichothecenes in liquid cultures and on inoculated wheat heads in the *tri12* mutants compared with PH-1. A significant reduction in disease caused by the *tri12* mutant was also reported, indicating that TRI12 is not only involved in self-protection but by enabling secretion of trichothecenes during host-pathogen interactions also contributes to pathogenicity. A *tri12* mutant was also reported for a *F. graminearum* NIV producer [99]. While toxin accumulation is significantly reduced in *tri12* mutants of NIV and DON chemotypes, the difference between *tri12* and wild-type *F. graminearum* is on the order of 10-20% [99], which is notably smaller than the 97% difference observed for the *F. sporotrichioides* mutant [33].

Efflux mechanisms have also been implicated in the response of other microorganisms to trichothecene toxins. *Saccharomyces cerevisiae* has been shown to tolerate DON at high concentrations; as much as 400 ppm is required for 50% growth retardation. Adam et al. [225] screened mutants for the pleiotropic drug resistance (PDR) genes in *S. cerevisiae* and determined that *PDR5* is involved in DON tolerance. PDR5 is a plasma membrane localized ABC transporter. Differential expression of ABC transporters were recently reported by Demissie et al. [226] as part of the *Clonostachys rosea* defense response to *F. graminearum*-spent medium, which contains secreted *Fusarium* metabolites. *C. rosea* is a biocontrol fungus that can control FHB in field settings, providing up to 58% reduction in disease index and a 21-33% reduction in DON [227,228]. At this time the actual function of these transporters is unknown, but it may provide protection of *C. rosea* against the effects of DON or drive the secretion of factors that inhibit *Fusarium* growth.

ABC transporters have also been implicated in trichothecene resistance in plants. Several ABC transporters were identified in differential gene expression and proteomics studies as part of the host reaction to *Fusarium* or DON treatments [219,229,230,231,232,233,234,235,236]. Walter et al. [235,236] identified an ABC transporter type C (ABCC) that is both *Fusarium* and DON-inducible. This gene was found to be regulated by the well-known resistance quantitative trait loci (QTL), *Fhb1*. Using virus-induced gene silencing in the Sumai-3 derived wheat line, CM82036, the authors determined that *TaABCC3.1* is involved in DON tolerance [235]. It should be noted that they also observed an interaction with the *TaABCC3* gene and grain development [235], and ABC transporter gene expression was also reported in a Treatment x Development interaction [237]. These transporters are evidently important components of the host-response, and while it is hypothesized that they transport DON out of the cytosol and into the vacuole, their exact contribution to resistance has yet to be verified.

### 4.3. Enzymatic Detoxification of Trichothecenes

**Scheme 7:**
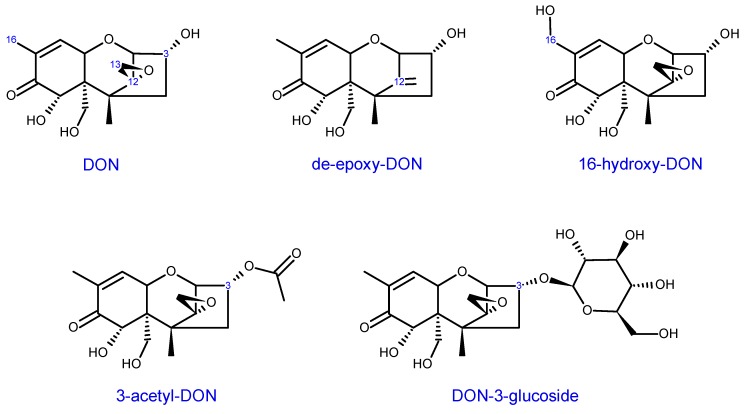
DON and detoxification products by de-epoxidation (de-epoxy DON; DOM-1), C_16_ hydroxylation (16-hydroxyl-DON; 16-HDON), C_3_ acetylation (3-acetyl-DON; 3-ADON), C_3_ glycosylation (DON-3-glucoside; D3G).

Several mechanisms of trichothecene detoxification have been identified in different organisms. Boutigny et al. [238] provide an excellent overview of known detoxification methods in planta. Among these is the glycosylation of DON at C_3_, yielding DON-3-glucoside (D3G) (Scheme 7), by the enzyme uridine diphosphate-glucosyltransfers (UGT) [203]. As described in Section 3.3, modification of the C_3_ hydroxyl groups tend to reduce or eliminate toxicity of DON and related trichothecenes. Differential expression of UGTs have been observed in the wheat and barley response to Fusarium infection or DON treatment [229,230,231,233,239,240,241]. He et al. [242] identified 179 putative UGTs encoded in the wheat genome. Among these, 59% and 69% were found to be up-regulated by F. graminearum treatments in an FHB susceptible cultivar, Annong 8455, two- and four-days post-inoculation. The authors also noted that inoculation with a DON non-producing F. graminearum mutant led to up-regulation of fewer UGTs. Transgenic expression of different UGTs have also been found to improve Fusarium resistance in different plants and tissues, and reduced DON or NIV accumulation in grains [243,244,245,246,247,248].

Another example of a DON detoxifying enzyme that functions by C_3_ modifications are the 3-O-acetyltransferase, encoded by the Fusarium TRI101 gene (Table 3). It is thought that this is another mechanism employed by the fungus to protect itself from toxicity [200]. Interestingly, 3-O-acetyltransferases have been identified in a number of trichothecene non-producing Fusaria, both functional TRI201 genes encoding 3-O-acetyltransferase and non-functional pseudo-genes have been reported [84,249,250]. While 3-ADON (Scheme 7) itself is generally non-toxic to plants, it is converted to DON in planta (*https://scabusa.org/pdfs/don_white-paper_6-07.pdf*), and as discussed in Section 3.3, exogenous applications of 3-acetyl-trichothecenes do not always display reduced phytotoxicity compared with applications of their non-acetylated counterparts. Nevertheless, transgenic expression of the F. graminearum or F. sporotrichioides TRI101 gene in planta can lead to improved FHB resistance and DON tolerance in some instances [251,252,253]. Alexander [254] provides a nice overview of the work on TRI101 engineering in plants. Interestingly, while TRI101 from F. graminearum and F. sporotrichioides are each capable of acetylating both Type A and Type B trichothecenes, kinetic assays by Garvey et al. [255] revealed that FgTRI101 is not only more efficient in its ability to acetylate DON and NIV compared with FsTRI101, but appears to be equally efficient at acetylating T-2 toxin.

While C_3_ modifications can reduce or eliminate toxicity of DON and related mycotoxins, these modifications are often reversed in the intestinal tract of mammals, which may be problematic if left undetected in food and feed. Acetylated DONs are also converted to DON during the baking process [256]. In any case, the simple fact that such modifications improve plant disease resistance generally translates into reduced toxin contamination.

De-epoxidation of trichothecenes is perhaps a more reliable mechanism of detoxification. While epoxide hydrolases are present in plant genomes, there is no evidence that these enzymes participate in trichothecene detoxification in planta. On the other hand, some microorganisms identified from soil [257], as well as fecal and intestinal microflora from diverse animal species [258,259,260], including humans [261], are capable of reducing the epoxide group of various trichothecenes. De-epoxy-DON (DOM-1) (Scheme 7) has also been reported in human excretory fluids [262]. There are also incidences where DOM-1 was not detected when human gut microflora was exposed to DON (e.g., [263]), or where DON was present in urinary samples (e.g., [264]). The reported differences among studies may reflect population differences or variances in experimental design and/or instrument sensitivities.

Another type of modification has been observed through the activities of cytochrome P450 enzymes. In 2012, Ito et al. [265] identified a cytochrome P450, DdnA from the aquatic bacteria, Sphingomonas sp. strain KSM1, capable of hydroxylating the C_16_ methyl group of DON, yielding a non-toxic product, 16-HDON (Scheme 7). Similar catabolic activities were observed against 3-ADON and NIV. In 2018, a wheat cytochrome P450, TaCYP72A, was reported to promote resistance against DON [266]. Virus-induced gene silencing of TaCYP72A resulted in increased sensitivity to DON-induced bleaching in the spikelets of the FHB resistant wheat line, CM82036.

### 4.4. Other Trichothecene Resistance Genes

Various genes have been reported to be differentially regulated by trichothecenes, some of which have been implicated in trichothecene resistance, but without a clear role in toxin degradation, efflux or ribosomal resistance. Gunupuru et al. [267] offers a review of DON resistance and describes in addition to those mechanisms discussed above, a number of genes reported in different expression studies investigating the effects of DON. For example, exogenous DON application in FHB resistant wheat induced expression or up-regulation of similar genes to those reported in response to DON-producing Fusaria, including receptor-like kinases, pathogenesis-related proteins, antioxidant enzymes such as glutathione-S-transferases and peroxidases, and enzymes in the phenylpropanoid pathway [219,268,269]. In some cases, DON-inducible genes are also expressed in susceptible wheat, where they occur earlier in resistant genotypes [219]. Similar observations with regards to temporal expression have been reported for Fusarium-inducible genes (for example [219,270,271]).

Many of the DON-induced genes are likely linked to general cellular responses that alleviate the effects of trichothecenes and these gene products may not interact directly with the toxins. For example, the up-regulation of antioxidant proteins could presumably reduce oxidative stress imposed by the toxins. In other cases, altered expression of cell signaling components might influence the expression of trichothecene efflux/detoxification genes, among other pathogen defenses. Various plant signaling hormones are found to regulate resistance or susceptibility to Fusarium [272,273,274,275,276,277,278,279,280,281,282,283]; meanwhile, expression of ABC transporters and UGTs were reported to be up-regulated by exogenous applications of jasmonic acid, and in the case of UGTs also by salicylic acid and the ethylene precursor 1-aminocylcopropane carboxylic acid [203,235].

Perochon et al. [284] identified a novel Sucrose Non-Fermenting Related Kinase (SnRK)-interacting protein involved in DON resistance, which they called Triticum aestivum Fusarium Resistance Orphan Gene (TaFROG). TaFROG expression was up-regulated by the F. graminearum strain GZ3639 (DON chemotype), but not by its trichothecene non-producing mutant, tri5. The putative orthologue was also similarly regulated in barley [285]. Analysis of transgenic lines overexpressing TaFROG and of virus-induced gene silenced lines, revealed improved resistance both to F. graminearum, as well as DON [284]. A TaFROG-interacting NAC transcription factor reported earlier this year was also shown to be involved in F. graminearum and DON resistance [286]. The mechanism of DON resistance has not been reported.

## 5. *Fusarium* Resistance to Limit Trichothecene Contamination of Food and Feed

### 5.1. Types and Forms of Fusarium Resistance

Trichothecene accumulation in stems and kernels of infected cereals are responsible for trichothecene contamination of food and contribute to the contamination of feed [21,22,23]. The most effective approach to preventing trichothecene contamination of cereal grains is to combine disease management strategies with the cultivation of varieties with high levels of FHB resistance. Disease management will not be discussed here, but various reviews are available on the subject [26,120,287,288,289,290,291,292]. Five types or forms of FHB resistance have been described, Type I to Type V (Table 4), which have also been numbered with Arabic numerals or alphabetically and are nicely summarized in Mesterházy [293]. In 1963, Schroeder and Christensen [294] defined Type I and Type II resistances as resistance to initial infection of the spike and resistance to spread of disease from spikelet to spikelet, respectively. Type III resistance is described as a resistance to kernel damage. Type IV resistance, or tolerance to trichothecene mycotoxins, was originally described by Wang and Miller in 1988 [295] according to the ability of some plants to tolerate the effects of 3-ADON. Miller et al. [296] had earlier described another form of resistance, according to the ability of some resistant cultivars to minimize DON accumulation either by degradation or by preventing its synthesis. This type of resistance (originally labelled Type III resistance [297]), was later identified as trichothecene resistance (Type V), and further subdivided by Boutigny et al. [238] into two classes based on the host’s ability to degrade or detoxify trichothecenes (Class 1) or to prevent their accumulation (Class 2).

In maize, two forms of resistance have been reported [27]. The first is silk-resistance, sometimes compared with Type I resistance in cereals, where the fungus is unable to penetrate the silk channel and is thereby prevented from accessing the kernels. The second is kernel-resistance, where the pathogen is unable to penetrate the cob and cannot then spread from one kernel to another. While cereal and maize inflorescence physiology differs, kernel resistance shares similarities to Type II resistance in cereals, where the fungus is unable to penetrate the rachis, thereby preventing spread from one spikelet to another.

### 5.2. Screening for Fusarium and Trichothecene Resistance in Cereals

Type I and II FHB disease resistances are the best characterized and the easiest to evaluate. Type I resistance quantifies resistance to initial infection expressed as a percentage of diseased spikes (disease incidence), whereas Type II resistance measures resistance to fungal spread within the spike mostly given as a percentage of disease spikelets within infected spikes (head severity). Often an ‘FHB index’ is calculated by multiplying the disease incidence × head severity/100 [120]. Under suitable environmental conditions where the temperature and humidity are favorable for disease, numerous additional factors can unduly influence the evaluation of incidence, for example some heads within a plant or plot may escape inoculation due to their position within the canopy or their developmental stage. Type II resistance can be more reliably determined and is measured by point inoculation, where spores are injected/pipetted into an individual floret or spike, and disease spread is reported as the number of diseased spikelets within 18–20 days after inoculation. Shaner [298] provides a valuable discussion on screening methods to evaluate different types of resistance and describes therein some of the challenges associated with screening for Type III to V resistance.

Detailed descriptions of inoculation methods are available from different sources [299,300,301,302,303]. Alternative methods to evaluate disease resistance have also been described that are designed to save time and/or enable high-throughput analyses (for example, [304,305]). Most of these involve analysis of different tissues of cereals such as seedlings or roots; for an excellent review of different inoculation methods in different tissues see Miedaner [306]. While the pathology and mechanisms of resistance in different organs are unlike those of FHB, these assays can sometimes serve as a valuable tool to test a hypothesis and/or identify candidate genes involved in resistance. For example, Wang et al. [307] investigated molecular aspects of resistance to FRR and determined that similar defense response genes are activated in the roots as observed in the spikes following FHB infection. Fusarium seedling blight, root and crown rots are different diseases of cereals caused by trichothecene-producing *Fusarium* species, and while they tend to affect plant survival and yield, they do not typically result in toxin contamination of grain. However, in the case of FCR, the infection can move up into the spike and result in DON contamination of seeds [23]. With the exception of FCR [308], these diseases do not have notable economic significance.

When assessing FHB, in addition to providing suitable environments for fungal infection, such as mist-irrigation in greenhouse and field settings to increase humidity [301], the influence of the growth stage is critical in disease assessment as this can influence interpretation of results. Spikelets inoculated prior to anthesis typically do not develop FHB symptoms. In barley, anthesis occurs within the boot, and as a result the spikes are not threatened by the disease pre-anthesis. When comparing inoculation in barley at different developmental stages after heading, McCallum and Tekauz observed no differences in disease response. Although, later infections may be symptomless while still accumulating mycotoxin [309]. In barley there is a low correlation between *Fusarium*-damaged kernel (FDK) and DON; FDK/DON correlations are even lower than that between FHB and DON. Thus, a grain sample that seems acceptable based on color, plumpness, and protein can carry high levels of DON [310,311]. In wheat the disease is most severe when spikes are inoculated at anthesis. Del Ponte et al. [312] compared disease severity, FDK and DON accumulation in the wheat cultivar Norm at six reproductive stages. Visible symptoms were most severe when inoculated at anthesis and decreased with inoculations at different stages of kernel development until the early dough stage. Meanwhile, DON occurred at all stages assessed, including the hard dough stage where kernel weight was unaffected and FDK symptoms were limited. These studies point to the importance of performing DON content evaluation in both wheat and barley.

Measurements of trichothecene accumulation can be carried out by immunodetection, such as enzyme-linked immunosorbent assays (ELISAs) that are commonly employed for high-throughput detection of DON and/or related mycotoxins. These methods are limited in that they provide information on individual toxins rather than a complete toxin profile, whereas mass spectrometry-based methods enable detection and quantification of all known toxins within an extract. More details on trichothecene quantification methodologies can be ascertained from the following articles: [26,313,314,315,316].

### 5.3. Trichothecences and FHB Pathogenicity

Trichothecenes are important components of aggressiveness and, in some hosts, are required for disease spread to occur. In wheat, when the fungus is unable to produce trichothecenes the disease is restricted to the inoculated spikelet(s) [317], except in cases where the mycelium spreads on the outside surface of the head [318]. *Fusarium* disruption mutants in the *TRI5* gene do not produce trichothecenes and are unable to gain access to the wheat rachis through infected spikelets [20,317,318,319,320]. In barley, where Type II resistance is inherent, different results have been reported. One study observed no differences in pathogenicity between three *tri5* mutant strains compared with their wild-type progenitors [20], while another reported that the *tri5* mutant was able to spread from spikelet to spikelet over the surface of barley spikes without penetrating the rachis [318]. Jansen et al. [319] used GFP-labelled GZ3639 and its *tri5* mutant and observed the spread of GZ3639 through the rachis of barley cultivar Chevron, but the *tri5* mutant was contained in the inoculated spikelet. Interestingly, in the case of maize, when comparing *tri5* mutants of NIV and DON chemotypes, it was observed that the mutant of an NIV chemotype was less aggressive than it’s wild-type, whereas no differences were observed between *tri5* mutants and their wild-type DON-producing counterparts [20]. These results demonstrate the complexity of the interaction between trichothecenes and mycotoxins with different cereals and their influence on pathogenicity.

### 5.4. Breeding for Low Trichothecence Content in Wheat and Barley Grains

Growing resistant cultivars is pivotal in *Fusarium* disease control and for the prevention of mycotoxin contamination, but resistance breeding is complicated by the quantitative nature of the trait involving multiple genes with small to medium effects and the interaction with environmental conditions [321]. Basically, resistance breeding relies on the available variation for the trait of interest and methods/tools to reliably measure or predict resistance levels and trichothecene contents in a breeding program. In both wheat and barley, genetic variation for FHB resistance is broad comprising ‘native’ and ‘exotic’ resistance sources. The difficulty for a breeder is to combine high yield and quality performance with resistance to other relevant diseases and pests including FHB. As a consequence, large numbers of breeding lines need to be screened in FHB disease nurseries to select superior lines favoring FHB disease assessments on the plants which are technically easier, faster and cheaper compared to direct mycotoxin quantifications.

In wheat, the relationship between visual disease evaluations in the field or on the harvested grains and DON content was investigated in a broad meta-analysis by Paul and coworkers [322]. They analyzed 163 studies resulting in overall positive and significant correlations, with FDK showing the strongest association with DON content (r = 0.73) followed by disease index (r = 0.62), severity (r = 0.53) and incidence (r = 0.52). The role of resistance in toxin control was also highlighted in a review [323] concluding similarly that breeding new cultivars with increased FHB resistance will result in reduced DON contamination, but moreover, reduces simultaneous levels of less prevalent and less frequently measured trichothecenes. For instance, consider the following field experiment where 190 winter wheat lines were inoculated with DON-producing *F. graminearum* and T-2/HT-2 toxin-producing *F. sporotrichioides* in separate trials and evaluated for symptom severity on the spikes and grains as well as DON and T-2/HT-2 toxin content. In this study, resistance measures correlated highly with DON and T-2/HT-2 toxin content within the trials and also with *F. graminearum* and *F. sporotrichioides* disease parameters across trials, but most noteworthy DON and T-2/HT-2 toxin contents were also associated (r = 0.80) demonstrating that indirect selection for low T-2/HT-2 toxin contents is feasible [323] and underlining the non-species-specific FHB resistance [324]. The effect of FHB resistance breeding in wheat on DON and its masked form D3G was discussed in a review by Lemmens et al. [325]. Several independent experiments revealed highly significant relationships between FHB symptoms on wheat heads, DON and D3G content indicating that selection of improved lines based on FHB symptoms or DON results in a concomitant reduction in D3G content [325,326,327].

Unlike wheat, a robust correlation between FDK and DON has not been reported in barley. However, Tucker et al. [328] recently reported a strong correlation between D3G content with DON and 3-ADON. These observations indicate that the selection of reduced DON content for development of improved barley breeding lines would likely result in lower D3G content.

QTL analysis unravels the genetic architecture of *Fusarium*/trichothecene resistance in a specific population in terms of the number and effect of QTL and elucidates the genetic basis of the trait associations. Twenty years ago, the first FHB resistance QTL were identified in wheat [329] and since then numerous QTL have been reported and summarized by Buerstmayr et al. [321] and by Buerstmayr (2019, review in preparation). The genetic basis of FHB resistance has also been studied through QTL analysis in barley and was reviewed by Kolb et al., 2001 [330], and later by Massman et al., 2011 [331].

Of particular interest in the context of this review are QTL (or genes) that confer resistance to trichothecenes and/or were found associated with reduced trichothecenes contents in the harvested grains of wheat and barley. Among the numerous FHB mapping studies in wheat, only 25 included DON measurements and provided further parameters for FHB resistance, for instance, the percentage of infected heads or spikelets, FDK, FHB severity or area under the disease progress curve (AUDPC) as a measure for overall field resistance or FHB index. These 25 studies identified 63 QTL linked with reduced DON contents of which 54 coincided with QTL for FHB parameters evaluated on the heads or grains, demonstrating a common genetic basis of FHB and DON resistance. Merely 9 QTL were detected exclusively for DON. Table S1 provides the complete list of wheat resistance QTL from experiments conducting DON measurements, whereas Table 5 excerpts major ‘DON resistance determinants’ including associations with loci controlling phenological and morphological traits.

The most prominent FHB resistance QTL in wheat, *Fhb1*, derived from Chinese germplasm, confers resistance to fungal spreading and reduces DON contents in grains as confirmed by many independent studies (Table 5). *Fhb1* governs ‘DON resistance in the narrow sense’; Lemmens et al. [336] showed by infiltrating pure toxin solution in wheat florets, that this locus enhances the hosts’ ability to detoxify DON. Lines carrying *Fhb1* conjugated almost all the applied DON into D3G whereas from those lacking *Fhb1* a high percentage of the infiltered DON was recovered. Thus, it was proposed that *Fhb1* either encodes or regulates a uridine diphosphate-glycosyltransferase (UGT). Fine-mapping revealed the complete contig sequence of the resistance locus, yet, no UGT was annotated [348,349]. A pore-forming toxin-like gene has been isolated and is responsible for resistance to fungal spreading at the locus [349]; however, recently two studies rebutted this finding, both identifying a critical deletion in the same gene encoding a histidine-rich calcium-binding protein as the causative mutation at *Fhb1*. Su et al. [350] concluded that the *Fhb1*-derived resistance is the result of a loss-of-function mutation, whereas Li et al. [351] demonstrated that the same deletion generates a gain of function [352]. Whether the histidine-rich calcium-binding protein also controls DON detoxification is yet unclear.

Besides *Fhb1*, other large effect FHB resistance QTL, *Qfhs.nau-2DL* [333,334], *Fhb2* [340], *Fhb5* [333,334,337,353], and *Fhb7AC* [339] also lead to reduced disease severity and reduced toxin content simultaneously. Morphological and phenological traits such as plant height, spike/flower morphology, and heading date affect fungal infection and spreading of the disease favoring taller genotypes with a lax spike type and high anther extrusion after anthesis. Furthermore differences in heading dates often result in disease escape predominantly because of environmental conditions [321]. Key determinants for these traits colocalize with QTL for FHB resistance and DON content, e.g., the height controlling loci *Rht-B1* and *Rht-D1 *[341,343,344], the *q* locus controlling spike type [346,347] and the *Ppd-D1* [332] and *Vrn-A1* [341,345] loci affect heading date and plant height. Common factors for anther extrusion and DON content were detected on chromosomes 2DLc [345,354], 7A*L* [354] and at the *Rht-B1* locus [341]. Whether all these relationships are due to pleiotropic effects or are caused by linked genes remains unknown. Few QTL were identified exclusively for DON which may indicate that plant genes exist which have a function in detoxification, but are not associated with FHB resistance in the narrow sense [354,355,356,357,358], although overlapping with QTL for plant height [355], anther extrusion [354] and heading date [358] was found for three of these loci. Also, a recent meta-QTL analysis of FHB resistance in wheat positioned the 63 QTL for DON resistance within 40 of the 65 generated meta-QTL mainly overlapping with Type I and II resistance QTL [359].

To date, most genetically mapped resistance QTL has no biological function assigned, but this has not affected their deployment in resistance breeding using phenotypic and/or genotypic selection. Molecular mapping studies yield markers linked with resistance QTL and enable selection of improved individuals based on genetic fingerprints. The so-called marker-assisted selection (MAS) is successfully applied to introgress well-characterized, large-effect QTL reducing FHB severities and DON contents [353,360,361,362]. Miedaner et al. [353] reported that marker-selected lines carrying the major resistance QTL *Fhb1* and *Qfhs.ifa-5A* reduced average DON content by 59 and 43% compared to their sister lines lacking the resistance alleles, and DON content was lowered by 79% in lines with combined resistance QTL alleles. However, many of the genes contributing to resistance have small effects, especially those derived from non-Chinese sources, unsuitable to track with few markers. The common practice to pyramid these by phenotypic selection can nowadays be accelerated by genomic selection, thereby estimating genome-wide marker effects in a phenotyped training population and predict genomic estimated breeding values of non-phenotyped individuals in a selection population [363]. The applicability of genomic selection for FHB and DON resistance breeding has been demonstrated in several studies [363,364,365]. Regarding DON content, both genomic-estimated breeding values and phenotypic selection would select superior lines that are encouraging from a breeding perspective, as genomic selection can save time and resources associated with phenotyping [363].

Relatively speaking, fewer FHB and DON QTL studies have been carried out in barley compared to wheat, with the majority of them being conducted in six-row barley. It was identified that resistance to FHB and DON accumulation are controlled by many QTL located on all seven barley chromosomes [330,331]. The detected QTL are often minor, environmentally specific, and associated with phenological and morphological traits. Taller stature, late heading, row-type, lax and nodding spike are commonly associated with FHB resistance [311]. Such unfavorable associations complicate introgression of FHB resistance into elite germplasm and are limiting the use of MAS in barley breeding programs.

Several examples of QTL from experiments that include DON measurements as well as associations with loci controlling phenological and morphological traits are presented in Table 6. The coincidence of DON QTL with FHB severity QTL mainly translates into lower DON accumulation being associated with reduced disease severity. However, there are some reports where lower DON QTL coincided with increased FHB severity QTL [366,367]. In addition, the case of QTL identified exclusively for DON and not coincident with FHB severity indicates that DON accumulation may not always be a pleiotropic effect of FHB QTL [366] and different genes might be responsible for it. The most commonly identified QTL for reducing DON concentration were detected on the chromosome 2H with the resistance allele contributed by resistant sources such as Chevron [367,368], CI 4196 [369], and Fredrickson [370]. These QTL have also been associated with a major heading date QTL and spike morphology controlled by *VRS1*. The recent work by Huang et al. [371] re-emphasizes the relationship between morphological characteristics and the FHB response and advocates that plant architecture and inflorescence traits must be absolutely considered when breeding barley for FHB resistance.

In 2013, an extensive germplasm screening was carried out by Huang et al. [376] on a global collection of 23,255 wild and cultivated accessions. This analysis identified only 78 FHB resistant or moderately resistant sources. These genotypes were further haplotyped with markers associated with consistently detected FHB QTL, located on chromosome 2H and 6H, from different resistant sources (Chevron, CI 4196, Fredrickson, etc.) by previous studies [331]. It was identified that the most common haplotype on all four QTL regions was one of the resistant source Chevron. Moreover, few other sources (cultivated or wild) with potentially novel alleles were identified based on their distinct haplotype patterns at these four QTL. That said, further mapping studies are required, which should also include DON measurements, before they can be deployed in the barley breeding programs.

Massman et al. [331] conducted a genome-wide association study using 768 advanced barley breeding lines and identified four QTL for FHB resistance and eight QTL for DON accumulation. DON concentration QTL was identified on every chromosome with four of them being in the same regions of chromosome 1H, 2H and 4H as those previously identified in bi-parental mapping populations [367,368,370,374]. Notably, a good portion of the QTL identified in this study are in many cases free of undesirable linkages makes them excellent candidates for MAS. Similarly, Mamo and Steffenson [377], when assessing a diverse collection of barley landraces from Ethiopia and Eritrea by genome-wide association, also found that the FHB resistance and DON concentration QTL identified were not significantly associated with heading date or plant height. Despite these new findings that could potentially enable MAS in barley, the authors [331] are proponents of the use of a more discursive marker selection strategy, such as genomic selection, as a way to amass the beneficial alleles in the barley breeding populations.

Sallam and Smith [378] compared genomic and phenotypic selection in five sets of spring six-row barley breeding lines for yield, FHB severity and DON concentration, and concluded that the use of genomic selection for these traits in barley breeding should result in gains similar to the ones obtained using phenotyping selection but in a shorter time frame and with a lower cost. Selection gains when using genomic selection for FHB and DON are also reported in two-row barley (James Tucker, Agriculture, and Agri-Food Canada, Brandon, personal communications). Moreover, Tiede and Smith [379] recently provided additional evidence for the effectiveness of genomic selection in six-row barley by demonstrating significant gains for these two unfavorably correlated quantitative traits, yield, and DON.

In addition, in vitro selection methods, which employ selection pressure for FHB and/or trichothecene resistant hexaploid wheat [380,381] and two-row [382,383] and six-row barley [384] have been established. With improved methodologies for green plantlet regeneration [385], this technology can now be employed on F_1_ hybrids, making this an attractive avenue to generate doubled haploid populations for FHB and trichothecene resistance breeding, which can also be combined with molecular genotyping approaches.

Resistance breeding programs need to find the optimal strategy within these complementary approaches according to their needs; ultimately, FHB resistant and productive cultivars are a sustainable, environmentally friendly, and economic way towards increasing food and feed safety and security.

## 6. Food and Feed Safety

Due to the significant health implications of trichothecene exposure in humans and animals, limits are placed on the allowance of trichothecenes in different food/feed products. North American and European guidelines for trichothecenes in human and animal food differ both in terms of the acceptable concentration limits and the specific affected commodities. These guidelines, summarized in Table 7 and Table 8, are provided by the Canadian Food Inspection Agency, the United States Food and Drug Administration, and the European Commission Regulations.

Regulations for DON and T-2 toxin content is generally less stringent for animal feed compared with processed cereals for human consumption. Humans and animals have the ability to metabolize and or excrete these toxins. For example, the toxins can be de-epoxidated and form conjugates with glucuronic acid, thereby increasing solubility which in turn facilitates secretion [386]. The carry-over of DON and other trichothecenes into meat products or milk harvested from farm animals that have been fed these toxins are considered negligible [386,387]. However, the sensitivity of animals to different trichothecenes can impact their health, lead to feed refusal and reduce their productivity, and there are species-specific sensitivities to the different types of trichothecenes. Trichothecenes can be teratogenic and cause malformations during fetal development [388,389,390], and a high stringency is placed on DON allowance in baby foods in Europe (200 ppb).

The *Fusarium* trichothecenes that contaminate cereal-based food and feed have had devastating impacts on human history. While our ability to monitor and limit their entry into the food/feed chain has improved over the years, they continue to persist, and this can have significant economic impacts as well health safety risks. The pathogens continue to evolve, and new emergent and masked mycotoxins are being identified [62,391]. In spite of the controls in place to regulate these toxins in food/feed, DON has been detected in the urine of different human populations [262], including children [264]. Thus, continued research efforts are needed to reduce trichothecene contamination of food and feed.

The body of work presented in this review summarizes the progress that has been made to date in trichothecene-related research in cereal crops. Producers of the Type B trichothecenes continue to dominate the landscape of FHB infected cereals around the world, with some geographic differences in the frequency of distribution of fungal genotypes/chemotypes. The Type A trichothecenes, T-2 and HT-2 toxin, which are more potent in mammalian systems than DON, may be increasing in frequency especially on oat and barley in some European countries. The identification of the NX-series of Type A trichothecenes found in some *F. graminearum* isolates endemic to North America, shows the need for a detailed description of fungal isolates from different regions of the world.

The trichothecenes owe their toxicity to their protein synthesis inhibiting activities in eukaryotic cells. These toxins occupy the A-site of the peptidyl transferase center, indicating that the trichothecenes are peptide elongation (E-type) inhibitors. Recent structural studies have allowed researchers to postulate that the structural rigidity on the tetrahydropyran core imposed by the epoxide ring is what makes the epoxide an essential element of toxicity, rather than the highly reactive nature of epoxides. Further to this, the hydroxyl group at C_3_ appears to further stabilize the structure, which may explain the disruptive effects of C_3_ acetylation or glycosylation on toxicity. More detailed structural analyses to better understand the toxicity mechanisms are needed to help us identify novel strategies to destabilize these molecules. Surveys of organisms that can metabolize trichothecenes can also lead to the development of remediation technologies. C_16_ hydroxylation is an example of a detoxification mechanism identified in both wheat and aquatic bacterium. Different host trichothecene detoxification mechanisms are effective in improving FHB disease resistance and in reducing toxin content. Breeders employ a variety of tools to select for improved resistance and low toxin content, and while tremendous progress has been made in improving resistance, high levels of resistance are still rare in elite cultivars and there is no immunity to this disease. It is imperative that research continues in this area to limit *Fusarium* diseases of cereals and to develop novel toxin remediation strategies in order to reduce the risk of human and livestock exposure to their toxins.

## Figures and Tables

**Table 1:** Substituent patterns of different Type A and B trichothecenes. OAc = *O-*acetyl, OIsoval = *O-*isovalerate.

	C_3_	C_4_	C_7_	C_8_	C_15_
***Type A***					
diacetoxyscirpenol (DAS)	-OH	-OAc	-H	-H	-OAc
trichodermin	-H	-OAc	-H	-H	-H
trichodermol	-H	-OH	-H	-H	-H
T-2 toxin	-OH	-OAc	-H	-OIsoval	-OAc
HT-2 toxin	-OH	-OH	-H	-OIsoval	-OAc
NX-2	-OAc	-H	-OH	-H	-OH
NX-3	-OH	-H	-OH	-H	-OH
***Type B***					
nivalenol (NIV)	-OH	-OH	-OH	=O	-OH
4-*O*-acetyl-NIV (4ANIV)	-OH	-OAc	-OH	=O	-OH
4-deoxy-nivalenol (DON)	-OH	-H	-OH	=O	-OH
3-*O*-acetyl-DON (3-ADON)	-OAc	-H	-OH	=O	-OH
15-*O*-acetyl-DON (15-ADON)	-OH	-H	-OH	=O	-OAc
trichothecin	-H	-OIsoval	-H	=O	-H

**Table 2:** Trichothecene pathway genes. Demarcation of the *Fusarium* core trichothecene and *TRI1-TRI16* clusters were reported by Brown et al. [68,69], and are reviewed in Alexander et al. [80].

Gene	Cluster	Gene Description	Activity
***Biosynthesis***			
***TRI1***	Tri1-Tri16	Cytochrome p450 monooxygenase	C_7_ hydroxylation [81] C_8_ hydroxylation [69,81,82]
***TRI3***	Core Tri	Acetyltransferase	C_15_ acetylation [83,84]
***TRI4***	Core Tri	Cytochrome p450 monooxygenase	C_2_ hydroxylation [76] C_3_ hydroxylation [77,78] C_11_ hydroxylation [77,78] C_12,13_ epoxidation [77,78]
***TRI5***	Core Tri	Sesquiterpene cyclase	Cyclization of farnesyl pyrophosphate; *TRICHODIENE SYNTHASE* [19,74,85]
***TRI7***	Core Tri	Acetyltransferase	C_4_ acetylation [86,87]
***TRI8***	Core Tri	Esterase	C_3_ deacetylation [88,89] C_15_ deacetylation [89]
***TRI11***	Core Tri	Cytochrome p450 monooxygenase	C_15_ hydroxylation [90]
***TRI13***	Core Tri	Cytochrome p450 monooxygenase	C_4_ hydroxylation [86,91]
***TRI16***	Tri1-Tri16	Acetyltransferase	C_8_ acetylation [69]
***TRI101***	None	Acetyltransferase	C_3_ acetylation [70,92,93]
***Transcription Factors***			
***TRI6***	Core Tri	Zinc-finger DNA binding protein	Regulation of *TRI* gene expression [94,95]
***TRI10***	Core Tri	Transcription factor	Regulation of *TRI* gene expression [96,97]
***TRI15***	None	Zinc-finger DNA binding protein	Negative regulation of trichothecene biosynthesis [71]
***Toxin Efflux***			
TRI12*	Core Tri	MFS transporter	Toxin efflux [33,98,99]
***Unknown***			
***TRI9***	Core Tri		
***TRI14***	Core Tri		Role in pathogenesis and trichothecene production [100]

* TRI12 has been described as TRI102 [101].

**Table 3:** Historically proposed a mechanism of protein translation inhibition of different trichothecenes.

Trichothecene	Translation Inhibition Type
DAS	I-type [186]
HT-2 toxin	I-type [186,187]
trichothecin	E-type [187], E-type [187]
T-2 toxin	I-type [186,187], T-type [189]
trichodermin	I-type [187], E-type [186,187,189,192], T-type [186,187,188,189,190,193]
trichodermol	E-type [187], T-type [187]
NIV	I-type [186,187]

**Table 4:** Types and forms of FHB and FEB resistance (table adapted from Foroud and Eudes [57]).

Type	Description
***FHB resistance***	
Type I	Resistance to initial infection [294]
Type II	Resistance to disease spread from one spikelet to another [294]
Type III	Resistance to kernel infection [293]
Type IV	Tolerance against FHB and trichothecenes [295]
Type V Class 1 Class 2	Resistance to trichothecenes [296] by Degradation or detoxification [238] by Preventing their accumulation [238]
***FEB resistance***	
Silk resistance	Resistance to silk penetration [27]
Kernel resistance	Resistance to cob penetration, preventing spread from one kernel to another [27]

**Table 5:** Major QTL associated with measures for FHB resistance and DON contents from studies including DON evaluations in wheat.

Reference	Chromosomal Allocation of QTL	Designated Gene/QTL	QTL of FHB Resistance Traits *	Coinciding Morpho/Phenological QTL	Source of Resistance Allele
Islam et al. [332]	2DS	*Ppd-D1*	type2, FHB sev, FDK, DON	flowering time, plant height	Truman
Jiang et al. [333,334]	2DL	*Qfhs.nau-2DL*	type2, DON		CJ 9306
Somers et al., Lemmens et al. Ma et al., Jiang et al., Yu et al., Jayatilake et al., and Zhao et al. [333,335,336,337,338,339,340]	3BS	*Fhb1*	type1, type2, FDK, DON, DON resistance		Sumai-3, CM-82036, Nyu Bai, Wangshiubai, CJ 9306, ND2710
He et al. [341]	4BS	*Rht-B1*	FHB sev, FDK, DON	plant height, anther extrusion	Ocoroni F86
Draeger et al., Liu et al., and Petersen et al. [342,343,344]	4DS	*Rht-D1*	type1, FHB sev, FHB index, AUDPC, FDK, DON	plant height	Arina, Ernie, Bess
Ma et al., and Jiang et al. [333,334,337]	5AS	*Fhb5*	type2, DON		Wangshuibai, CJ 9306
He et al. [341,345]	5AL	*Vrn-A1*	type2, FHB sev, FHB index, FDK, DON	heading date, plant height	Soru#1 synthetic hexaploid wheat, Ivan/Soru#2
Chu et al., and Zhao et al. [346,347]	5AL	*q*	type2, FHB sev, FDK, DON	plant height, spelt type	PI277012
Zhao et al. [340]	6B	*Fhb2*	type2, DON		ND2710
Jayatilake et al. [339]	7A	*Fhb7AC*	type2, FDK, DON		Sumai-3

* DON-Deoxynivalenol content; FDK-Fusarium damaged kernel; FHB index-percentage of infected spikes*percentage of infected spikelets* 0.01; FHB sev- FHB severity; type1-resistance to initial infection; type2-resistance to fungal spread within the spike.

**Table 6:** Examples of QTL associated with measures for DON contents and FHB resistance from bi-parental mapping studies including DON evaluations in barley.

Reference	Chromosomal Allocation of QTL*	Designated QTL	QTL of FHB Resistance Traits**	Coinciding Morpho/Phenological QTL	Source of Resistance Allele
de la Penna et al., and Ma et al. [367,368]	1H	NA	DON, FHB sev^, KD; DON	heading date, plant height; number of nodes per centimeter of rachis	Chevron
Canci et al., Dahleen et al., de la Penna et al., Horsley et al., Ma et al., Mesfin et al., and Zhu et al. [367,368,369,370,372,373,374]	2H (bin 8 to 10)	*Qrgz-2H-8, Qrgz-2H-10*	DON, FHB sev, KD	lateral floret size, heading date, plant height, rachis node number, seeds per inflorescence, spike type	CI 4196, Chevron, CMB643 (Azafran), Fredrickson, Zhedar 2
Horsley et al., and Zhu et al. [369,374]	4H (bin 2 and 4)	*QDON-4H-2*; NA	DON; DON, FHB sev	NA; seeds per inflorescence	CI 4196; Gobernadora (Zhenmai-1)
Dahleen et al. [373]	6H	NA	DON, FHB sev	heading date	Zhedar 2
Ma et al. [368]	7H	NA	DON, FHB sev	heading date, plant height,	Chevron

* Bin location as determined from Steptoe x Morex bin map [375]; ** DON-Deoxynivalenol accumulation/concentration; FHB sev- FHB severity; KD-kernel discoloration; NA-not applicable; ^increased FHB severity.

**Table 7:** DON allowance in food and feed.

**Food products**	
Finished wheat products for consumption by humans	1000 ppb (USA)
Uncleaned soft wheat for human consumption	2000 ppb (Canada)
Unprocessed cereals (excluding durum wheat, oats, and maize)	1250 ppb (Europe)
Unprocessed durum wheat and oats	1750 ppb (Europe)
Unprocessed maize	1750 ppb (Europe)
Cereal flour, maize flour, maize, grits, and maize meal	750 ppb (Europe)
Bread, biscuits, pastries, cereal snacks and breakfast cereals	500 ppb (Europe)
Dry pasta	750 ppb (Europe)
Processed cereal based baby and infant food	200 ppb (Europe)
**Feed**	
Grains and grain by-products destined for ruminating beef and feedlot cattle older than 4 months and for chickens	10 ppm (Europe)
Grains and grain by-products destined for ruminating beef and feedlot cattle older than 4 months and chickens (not exceeding 50% of the cattle or chicken total diet)	10 ppm (USA)
Grains and grain by-products destined for ruminating beef and feedlot cattle older than 4 months and chickens (not exceeding 50% of the cattle or chicken total diet)	5 ppm (Canada)
Grain and grain by-products destined for swine	5 ppm (Europe)
Grain and grain by-products destined for swine	5 ppm (USA)
Grain and grain by-products destined for swine	1 ppm (Canada)
Grain and grain by-products destined for other animals	5 ppm (Europe)
Grains and grain by-products (not exceeding 40% of the diet) for young calves, & lactating dairy animals	1 ppm (Canada)
Grains and grain by-products (not exceeding 40% of the diet) for young calves, & lactating dairy animals	5 ppm (USA)
Cereals and cereal products with the exception of maize by-products	8 ppm (Europe)
Maize by-products	12 ppm (Europe)
Complementary and complete feeding stuffs	5 ppm (Europe)
Complementary and complete feeding stuffs for pigs	0.9 ppm (Europe)
Complementary and complete feeding stuffs for calves (<4 months), lambs, kids and dogs	2 ppm (Europe)

**Table 8:** T-2 toxin and HT-2 toxin allowance in food and feed.

**Unprocessed cereals**	
Barley (including malting barley) and maize	200 ppb
Oats (with husk)	1000 ppb
Wheat, rye and other cereals	100 ppb
**Cereal grains for direct human consumption**	
Oats	200 ppb
Maize	100 ppb
Other Cereals	50 ppb
**Cereal products for human consumption**	
Oat bran and flaked oats	200 ppb
Cereal bran except oat bran, oat milling products other than oat bran and flaked oats and maize milling products	100 ppb
Other cereal milling products	50 ppb
Breakfast cereals including formed cereal flakes	75 ppb
**Cereal products for feed and compound feed**	
Oat milling products (husks)	2000 ppb (Europe)
Other cereal products	500 ppb (Europe)
Compound feed, with the exception of feed for cats	250 ppb (Europe)
Compound feed for cats	50 ppb (Europe)
Diets for cattle and poultry	0.1 ppm (Canada)
Diets for dairy animals	0.025 ppm (Canada)

## Supplementary Materials

The following are available online at https://www.mdpi.com/2072-6651/11/11/634/s1, Table S1: QTL for components of FHB resistance in wheat including DON.
